# Supplementary material for: Enzymatic Hydrolysis of Tenebrio molitor (Mealworm) Using Nuruk Extract Concentrate and an Evaluation of Its Nutritional, Functional, and Sensory Properties
Source: Foods. 2023 May 29;12(11):2188. doi: 10.3390/foods12112188 (PMC10252857; doi:10.3390/foods12112188)
Supplement: Supplementary file 1 [file foods-12-02188-s001.zip › foods-2384197-supplementary.pdf]

Supplementary materials

Table S1. Total amino acid composition of whole and defatted mealworm in g/100g of protein and compared with the FAO/WHO standard protein (2001)

AA (g/100g of protein)	Whole Mealworm	Defatted Mealworm	Reference (FAO/WHO 2001)
Essential AA			
His	3.49 ± 0.13	3.79 ± 0.06	1.5
Thr	4.09 ± 0.16	4.04 ± 0.08	2.3
Lys	5.89 ± 0.29	4.09 ± 0.21	4.5
Tyr	8.70 ± 0.33	9.32 ± 0.24	7.5
Val	6.82 ± 0.26	6.15 ± 0.16	3.9
Ilu	4.51 ± 0.17	4.15 ± 0.10	3
Leu	7.68 ± 0.31	7.24 ± 0.17	6.3
Phe	4.21 ± 0.15	4.49 ± 0.08	
Met	1.93 ± 0.06	1.35 ± 0.03	
Phe + Tyr	12.91 ± 0.48	13.81 ± 0.32	
Cys + Met	2.10 ± 0.09	1.69 ± 0.05	2.2
Non Essential AA			
Ser	4.65 ± 0.18	4.80 ± 0.09	
Arg	5.74 ± 0.20	5.99 ± 0.07	
Gly	5.53 ± 0.21	5.80 ± 0.07	
Asp	8.49 ± 0.32	6.14 ± 0.20	
Glu	12.83 ± 0.52	19.02 ± 0.55	
Ala	7.52 ± 0.31	6.92 ± 0.20	
Pro	7.46 ± 0.30	6.36 ± 0.15	
Cys	0.17 ± 0.03	0.34 ± 0.02	

Table S2: IC_{50} in mg/ml of ABTS and ACE inhibition

No	MW/Hydrolysate	ABTS Inhibition IC_{50} mg/ml	ACE inhibition IC_{50} mg/ml
1	MW-DF	11.51 ± 0.11	0.53 ± 0.01
2	MW-NEC	3.07 ± 0.03	0.15 ± 0.00
3	MW-A	2.73 ± 0.02	0.05 ± 00
5	MW-F	3.25 ± 0.01	0.24 ± 0.01

Table S3: Correlation matrix (Pearson) for biochemical/peptide composition and bioactivities and sensory properties

Variables	EAA	TAA	Sour	≥ 670 kDa	Salt	6.7 - 1.7 kDa	NEAA	ABTS IC_{50}	ACE IC_{50}	Sweet	≤ 1kDa	Bitter	Umami
EAA	**1**	0.870	**-0.951**	0.758	0.664	0.355	0.712	-0.637	-0.350	0.586	0.107	-0.005	-0.024
TAA	0.870	**1**	-0.925	0.890	0.766	0.684	**0.966**	-0.820	-0.640	0.588	0.221	0.047	0.105
Sour	**-0.951**	-0.925	**1**	-0.921	-0.860	-0.617	-0.816	0.843	0.620	-0.777	-0.381	-0.252	0.201
≥ 670 kDa	0.758	0.890	-0.921	**1**	**0.974**	0.872	0.868	**-0.985**	-0.876	0.880	0.639	0.498	-0.339
Salt	0.664	0.766	-0.860	**0.974**	**1**	0.875	0.741	**-0.983**	-0.906	**0.961**	0.787	0.676	-0.539
6.7 - 1.7 kDa	0.355	0.684	-0.617	0.872	0.875	**1**	0.788	-0.936	**-0.992**	0.760	0.755	0.618	-0.338
NEAA	0.712	**0.966**	-0.816	0.868	0.741	0.788	**1**	-0.833	-0.727	0.529	0.258	0.070	0.162
ABTS IC_{50}	-0.637	-0.820	0.843	**-0.985**	**-0.983**	-0.936	-0.833	**1**	0.945	-0.896	-0.736	-0.601	0.409
ACE IC_{50}	-0.350	-0.640	0.620	-0.876	-0.906	**-0.992**	-0.727	0.945	**1**	-0.825	-0.831	-0.710	0.457
Sweet	0.586	0.588	-0.777	0.880	**0.961**	0.760	0.529	-0.896	-0.825	**1**	0.867	0.804	-0.742
≤ 1k Da	0.107	0.221	-0.381	0.639	0.787	0.755	0.258	-0.736	-0.831	0.867	**1**	**0.981**	-0.864
Bitter	-0.005	0.047	-0.252	0.498	0.676	0.618	0.070	-0.601	-0.710	0.804	**0.981**	**1**	-0.934
Umami	-0.024	0.105	0.201	-0.339	-0.539	-0.338	0.162	0.409	0.457	-0.742	-0.864	-0.934	**1**

Values in bold are different from 0 with a significance level alpha=0.05

ABTS and ACE inhibition were in mg/mL

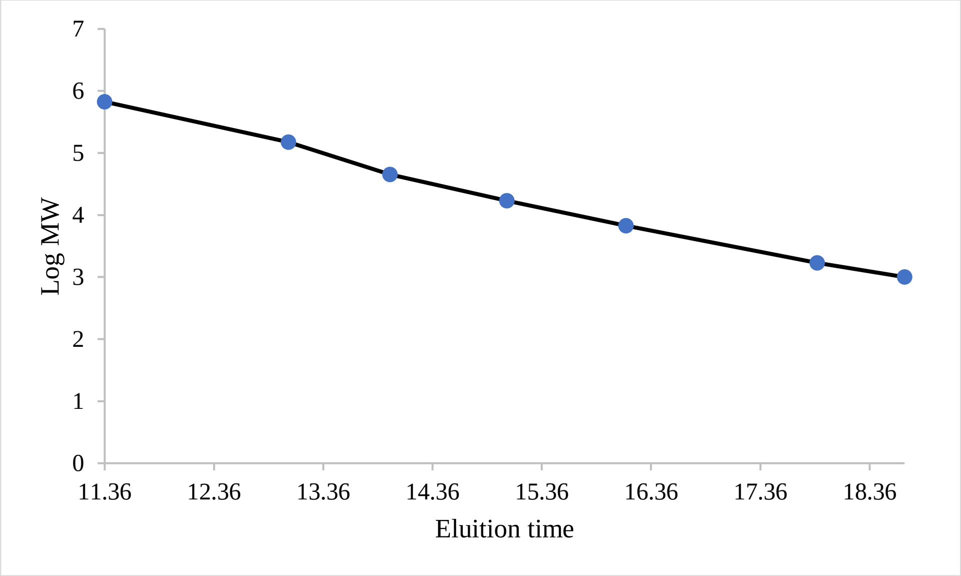

Figure S1. GPC calibration plot

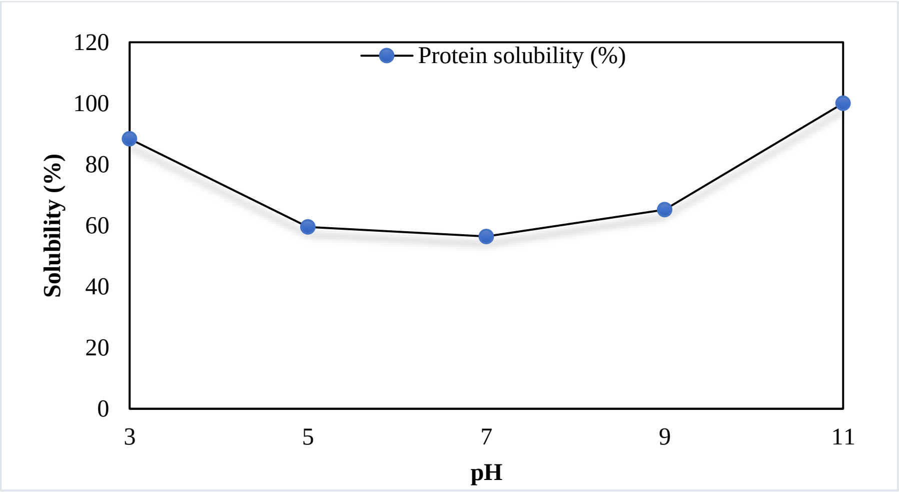

Figure S2. Solubility of defatted mealworm protein at different pH
